# Congenital anomalies in newborns to women employed in jobs with frequent exposure to organic solvents - a register-based prospective study

**DOI:** 10.1186/1471-2393-11-83

**Published:** 2011-10-27

**Authors:** Arild Vaktskjold, Ljudmila V Talykova, Evert Nieboer

**Affiliations:** 1 Helse UMB, Institutt for husdyr og akvakulturvitenskap, Universitetet for miljø- og biovitenskap, Ås, Norway; 2 Nordiske høyskolen for folkehelsevitenskap, Gőteborg, Sweden [a subsidiary of the Nordic Council of Ministers], Denmark; 3 Kola Research Laboratory for Occupational Health, Kirovsk, Russia; 4 Department of Biochemistry and Biomedical Sciences, McMaster University, Hamilton, Canada; 5 Institute of Community Medicine, University of Tromsø, Norway

## Abstract

**Background:**

The foetal effects of occupational exposure to organic solvents in pregnancy are still unclear. Our aim was to study the risk of non-chromosomal congenital anomalies at birth in a well-defined population of singletons born to women employed as painters and spoolers in early pregnancy, compared to women in non-hazardous occupations.

**Method:**

The study population for this prospective cohort study was singleton newborns delivered to working mothers in the industrial community of Mončegorsk in the period 1973-2005. Occupational information and characteristics of the women and their newborns was obtained from the local population-based birth register.

**Results:**

The 597 women employed as painters, painter-plasterers or spoolers had 712 singleton births, whereof 31 (4.4%) were perinatally diagnosed with 37 malformations. Among the 10 561 newborns in the group classified as non-exposed, 397 (3.9%) had one or more malformations. The overall prevalence in the exposed group was 520/10 000 births [95% confidence limits (CL): 476, 564], and 436/10 000 births (95% CL: 396, 476) in the unexposed. Adjusted for young maternal age, smoking during pregnancy, maternal congenital malformation and year of birth, the odds ratio (OR) was 1.24 (95% CL: 0.85, 1.82); for multiple anomalies it was 1.54 (95% CL: 0.66, 3.59).

The largest organ-system specific difference in prevalence between the two groups was observed for malformations of the circulatory system: 112/10 000 (95% CL: 35, 190) in the exposed group, and 42/10 000 (95% CL: 29, 54) in the unexposed, with an adjusted OR of 2.03 (95% CL: 0.85, 4.84). The adjusted ORs for malformations of the genital organs and musculoskeletal system were 2.24 (95% CI: 0.95, 5.31) and 1.12 (95% CI: (0.62, 2.02), respectively.

**Conclusion:**

There appeared to be a higher risk of malformations of the circulatory system and genital organs at birth among newborns to women in occupations with organic solvent exposure during early pregnancy (predominantly employed as painters). However, the findings were not statistically conclusive. Considering that these two categories of malformations are not readily diagnosed perinatally, the difference in prevalence between the exposed and unexposed may have been underestimated.

## Background

Organic solvents are widely used in industry and exposure is common for workers in manufacturing and processing operations. Many organic solvents are known teratogens and embryotoxins for laboratory animals [1], and solvent abuse in pregnancy is associated with an increased risk of major foetal anomalies [2]. However, the epidemiological evidence concerning developmental effects in humans due to maternal exposure in occupational settings is still weak and ambiguous. Literature reviews have suggested an association between high exposure to some organic solvents and the risk of foetal loss [3,4]. However, the findings concerning the impact of maternal occupational exposure to organic solvents on the risk of congenital anomalies have not been consistent [3,5-10]. Studying this issue is complicated by the fact that often exposure involves a mixture of solvents and is therefore difficult to classify. Currently, ingestion of ethyl alcohol is the only single organic-solvent exposure with well-documented teratogenic effects in humans [11-13].

Due to the challenge of capturing a large number of outcomes, published studies rarely have a prospective cohort or controlled study design [14]. This inherent weakness and the above mentioned classification difficulty add to the likelihood of publication bias and null findings. Studies with a case-control design have reported associations between maternal organic-solvent exposure and being born with oral clefts, digestive anomalies, multiple anomalies, gastroschisis, and with neural tube defects [15-21].

In Russia, the proportion women working in industry has traditionally been high. In the borough of Mončegorsk, located in the far north-west of the Russian Federation (RF), the main employer for women has been the local nickel refinery and associated operations. Some of the jobs, as in other enterprises in the community, involved working with organic solvents on a daily basis. Further, all births in the borough since 1973 have been systematically registered retrospectively [22]. In the present study we made use of this established birth register.

The aim of this register-based prospective study was to assess the risk of non-chromosomal congenital anomalies for singleton newborns to women employed as painters and spoolers, which are jobs that have been related to a relatively high exposure to organic solvents on a frequent basis.

## Methods

### Context

Mončegorsk had 66 200 inhabitants in 1995; a number that fell below 60 000 during the years that followed. The nickel refinery began its operation in 1938, and ran 24 hours a day with three work shifts. The refinery's operation and the refining processes in the various departments [23,24] have been described previously, as well as the employment placement of the delivering population in Mončegorsk [25].

Three types of jobs commonly held by women in Mončegorsk were of particular concern in terms of a high likelihood of regular exposure to organic solvents, namely painters, painter-plasterers and spoolers. The spoolers used acetone, benzene, toluene and xylene for removing lacquer from engine coils, while the painters and painter-plasterers were exposed to white spirit (Stoddard solvent), acetone, toluene, xylene, and/or benzene used as cleaners and thinners of oil-based paints in their daily work. The last 3 solvents are aromatic hydrocarbons; acetone is not, and Stoddard solvent contains 10-20% aromatic hydrocarbons [26].

For the mentioned workers, inhalation is the major route of exposure. Air monitoring of the spoolers' work zones carried out by the nickel plant's Control and Analytical Centre in the year 2000 revealed that the mean concentrations of acetone, toluene and xylene were about three times higher than the exposure limits in the RF. The limits were 200, 150 and 50 mg/m^3 ^according to the federal Kola Research Laboratory of Occupational Health in the nearby town of Kirovsk, who received mandatory reports from the nickel refinery on health and safety issues.

For the study time frame, the prenatal care in the RF was without service fees, and pregnant women were advised to visit a gynaecologist before 12 weeks of pregnancy. Women doing heavy or risk-related work were to be transferred to another task on confirmation of the pregnancy. After 30 weeks, the pregnant women were obliged by law to take a work leave. A pregnancy could be terminated for medical reasons (including severe foetal defects) until the 22^nd ^week. The maternal care and maternal benefits, the definitions of a live birth, stillbirth and spontaneous abortion, as well as the regulations for induced abortion in the RF, have previously been described in some detail [22].

### Exposure and outcome data

The source of the data in this investigation was the birth register in Mončegorsk, which was set up for research purposes and the first of its kind in the RF. All births in the period 1973-2005 were registered systematically. In addition to the delivery records, register information was also obtained from the maternal gynaecological records (including case histories). A comprehensive account of the history, data collection and data quality of the register has been published [22]. In total, 26 846 births were registered, of which 26 415 were singleton.

Information on congenital anomalies in the register includes up to 5 diagnoses per newborn (diagnosed perinatally). Both the ICD-10 code and the diagnosis spelled out in words are registered in adjacent fields. The diagnoses were entered in the order they were cited in the birth records.

Details about the delivering women's job functions and employer at the onset of pregnancy were registered by a numeric code, as well as with a precise word descriptor. The recorded job functions were employed to classify the newborns (live- and stillborn) as exposed or non-exposed to organic solvents during early pregnancy.

### Study population and sample

The study population consisted of singleton newborns with at least 28 weeks of gestation, born of employed women and delivered in Mončegorsk in the period 1973-2005. The newborn was excluded from the study if: the source chart containing diagnoses at birth was missing; the interpretation of the recorded diagnosis was uncertain; or the diagnosis was foetal alcohol syndrome or a chromosomal anomaly (191 excluded, of whom 3 mothers were exposed to solvents). The exposed study group encompassed 712 singleton births by 597 different women employed as spoolers, painter-plasterers or painters. The non-exposed group included all singleton newborns to women employed in occupations without solvent exposure (n = 10 561). Not included were 14 951 neonates whose mothers were not employed, had occasional or low exposure to solvents^a^, or in jobs involving exposure to other potential teratogenic hazards^b ^[27].

Given a prevalence of malformations of 4.0 percent, the study had a statistical power of 86 percent to detect an overall relative-risk difference of 1.6 between the exposed and unexposed groups.

### Analyses

Exposure to organic solvents was defined as a dichotomous variable, exposed or non-exposed. The risk of congenital anomalies was analysed both by grouping all non-chromosomal malformations and deformations into categories as defined in the ICD-10 classification system, and by pooling them (ICD-10: Q00-89, except Q86.0). Congenital dislocation of hip (Q65) was not included. More than one malformation per newborn was included, and the organ-group specific prevalence was estimated in each exposure group.

Multiple logistic regression analyses were used to estimate the adjusted odds ratios (OR) and their 95% Wald confidence limits (CL) for a congenital malformation, using the unexposed study group as the reference group. In addition, newborns with multiple malformations were analysed separately.

The following *a priori *selected confounding factors were included in the models for adjustment: maternal age <18 years (yes/no); maternal congenital malformation (yes/no); evidence of heavy smoking during pregnancy, as noted in the gynaecological records (yes/no); and year of birth (categorised in 3-year intervals).

This study conforms to the Helsinki Declaration and to local legislation. The birth register and its use for epidemiological studies were approved by the Regional Ethics Committee of Northern Norway and the Regional Health Administration of Murmanskaja Oblast.

## Results

The proportions of births that were primipara and by women younger than 18 years were slightly higher in the unexposed group. The other maternal characteristics were similar between the two groups, and to the population as a whole (details are outlined in Table 1). In the exposed group, there were 24 births by women working as spoolers, 187 by painter-plasterers, and 501 by painters. None of the newborns of spoolers were diagnosed with a malformation.

**Table 1:** Characteristics of the solvent exposed and unexposed study groups, and the source population

	Population^1,2 ^	Exposed^1^	Unexposed^1^
Number of observations	26 415	712	10 561

Births with maternal age <18 years (%)	2.1	1.1	2.6

Maternal alcohol abuse in pregnancy (%)	0.5	0.7	0.3

Maternal tobacco smoking in pregnancy (%)	1.9	1.7	1.3

Maternal malformation (%)	1.2	1.0	1.4

Maternal diabetes mellitus	0.1	0.0	0.1

Mean first antenatal visit in weeks (SD)^3^	14.2 (7.9)	14.1 (7.6)	13.6 (7.1)

Primiparous births (%)	52.4	48.3	46.8

Stillborn (%)	1.0	1.1	0.8

Mean maternal age in years (SD)^3^	25.2 (5.1)	24.3 (4.6)	25.1 (5.1)

^1 ^More than one birth per woman included. ^2 ^All singleton births in the source population. ^3 ^SD = standard deviation

Group-specific frequency and prevalence of congenital anomalies (grouped by body organ or system) in the exposed and unexposed groups and the whole birth population are presented in Table 2. In the unexposed group, 460 malformations were diagnosed in 397 newborns (3.8%) compared to 37 malformations in 31 newborns in the exposed group (4.4%). The overall prevalence was 520/10 000 births (95% CL: 476, 564) in the exposed group and 436/10 000 births (95% CL: 396, 476) in the unexposed. The overall adjusted OR was 1.24 (95% CL: 0.85, 1.82). The largest organ-system difference in prevalence between the two groups was observed for malformations of the circulatory system; 42/10 000 (95% CL: 29, 54) in the unexposed group and 112/10 000 (95% CL: 35, 190) in the exposed. The adjusted OR for this category of malformations was 2.03 (95% CL: 0.85, 4.84). The organ-group specific ORs are provided in Table 3. For multiple anomalies, the adjusted OR was 1.54 (95% CL: 0.66, 3.59). The Hosmer-Lemeshow test indicated good model fit for the multiple analyses, and none of the covariates had to be removed from the pooled model because of co-linearity.

**Table 2:** The organ-group specific frequency (freq) and prevalence (prev) of congenital anomalies in the solvent-exposed and unexposed study groups, and in the source population minus exclusions

	Population^1^		Unexposed		Exposed	
Organ group (ICD-10 code)	Freq	Prev^2^	Freq	Prev^2^	Freq	Prev^2^

Nervous system (Q00-07)	110	42	41	39	0	NA^3^

Eye, ear, face and neck (Q10-18)	40	15	11	10	0	NA^3^

Circulatory system (Q20-28)	120	46	44	42	8	112

Respiratory system (Q30-34)	47	18	19	18	0	NA^3^

Digestive system (Q35-45)	94	36	30	28	3	42

Genital organs (Q50-56)	123	47	47	45	6	84

Urinary system (Q60-64)	116	44	45	43	3	42

Musculoskeletal system (Q66-79)	457	174	178	169	13	183

Other malformations (Q80-89)^4^	116	44	45	43	4	56

Total	1223	465	460	436	37	520

^1 ^All singleton births in the source population except newborns with foetal alcohol syndrome and uncertain or missing diagnoses; n = 26 255. ^2 ^Prevalence per 10 000 births. ^3 ^Not applicable. ^4 ^Q86.0 not included.

**Table 3:** The odds ratios (OR) for selected organ-group specific malformations

Organ group (ICD-10)	OR (95% confidence limits)
Nervous system (Q00-07)	NA^1^

Eye, ear, face and neck (Q10-18)	NA^1^

Circulatory system (Q20-28)	2.03 (0.85, 4.84)^2^

Respiratory system (Q30-34)	NA^1^

Digestive system (Q35-45)	1.65 (0.50, 5.46)^3^

Genital organs (Q50-56)	2.24 (0.95, 5.31)^2^

Urinary system (Q60-64)	1.06 (0.33, 3.43)^3^

Musculoskeletal system (Q66-79)	1.12 (0.62, 2.02)^3^

Overall (Q00-89)^4^	1.24 (0.85, 1.82)^2^

^1 ^Not applicable. ^2 ^Adjusted for maternal age <18 years, maternal malformation, smoking, and year of birth. ^3 ^Not adjusted for co-variates due to poor model fit. ^4 ^Q65 and Q86.0 not included.

## Discussion

The prevalence of malformations appeared to be higher among newborns to women employed as painters in early pregnancy; mainly due to the higher proportions of malformations in the circulatory system and genital organs. However, the number of exposed was not large enough to detect a statistical difference in risk between the exposed and unexposed groups, and adjusting for the included risk factors did not change that finding. Nevertheless, the point estimate of the overall risk had a higher precision in the present study than that reported in previously published cohort studies [6,8,9].

During the embryonic and early foetal periods, susceptibility to induction of malformations of the circulatory, genital and musculoskeletal systems may be considered high. Animal studies with benzene, toluene and xylene, but not acetone and Stoddard solvent, suggest only the occurrence of skeletal malformations [28-30]. Defects of the circulatory and the genital systems were not reported, and perhaps were outside the scope of the experiments conducted. From the mechanism-of-action perspective, it is pertinent that metabolic activation of the three aromatic solvents is known to generate similar reactive species that are capable of causing oxidative damage and they thereby possess teratogenic potential [26].

Few studies of the association between organic solvent exposure and specific malformations or groups of malformations are available that focus on occupational exposures to aromatic hydrocarbons during pregnancy. A case-control study by McDonald et al. (1987) found an 8-fold increase in overall risk to offspring of women exposed to aromatic solvents, mainly toluene. The increased risk primarily concerned gastrointestinal and renal-urinary defects [31]. Exposure to toluene was also suggested as the explanation of the higher odds of oral cleft in newborns to women working in the leather and shoe industry [32,33]. An increased risk of oral clefts has also been associated with several different classes of organic solvents [16,18]. A *post-hoc *analysis of oral clefts in our study gave an adjusted OR of 1.9 (95% CI: 0.4-8.4), but there were only two such outcomes in the exposed group.

In the few prospective studies published, only the effect of exposure to a mixture of organic solvents has been examined. Both overall associations [5-7] and their absence [8,10] have been reported. The finding of an overall association with non-chromosomal malformations, primarily reflecting the occurrence of oro-facial clefts and urinary, male genital [7] and musculoskeletal malformations [5], support our results (urinary malformations excluded). However, it was unexpected that malformations of the circulatory system appeared to have the largest difference in prevalence between the exposed and unexposed in our study. Most of the circulatory malformations in the exposed group were of the aortic and mitral valves (Q23). In case-control studies, ORs for cardiovascular malformations among the solvent-exposed subjects of 1.3 (95% CL: 0.8-2.2) [29] and 1.0 [17] have been reported. Furthermore, studies involving pregnant solvent abusers, who expose their foetuses to very high concentrations in each binge episode, have reported strong teratogenic outcomes. The latter had features of foetal alcohol syndrome (i.e., mainly microcephaly and craniofacial abnormalities) [2,34].

Prevalences of malformations in different populations are not readily comparable [35,36]. However, the overall rate in the study population was similar to that of Norway [37,38], which suggests that the general perinatal detection of malformations at birth in Mončegorsk reflects comparable identification protocols. Some malformations, such as those of the genital organs and the circulatory systems, may be misclassified or under-ascertained before discharge from the birth clinic [35,39,40]. This would have influenced the estimated prevalences, but it is unlikely that the diagnostic procedures and specifications, or recording of cases in the delivery records, would have been differential between the exposed and unexposed. The malformations of the heart and other circulatory anomalies constituted 21 percent of all malformations in the exposed group, compared to 11 percent in the unexposed. Consequently, it is unlikely that the explanation of this difference was a differential likelihood of being diagnosed during the stay in the clinic. On the other hand if occupational organic-solvent exposure elevated the risk of malformations that inherently are difficult to detect in the early neonatal period, and/or of malformations that lead to termination of pregnancy after a prenatal diagnosis, the overall relative risk between the exposed and unexposed in our study would have been underestimated.

Several studies have reported an elevated risk of spontaneous abortions among women exposed to organic solvents [12]. If an increased incidence of anomalies promotes such an outcome, or terminations occur due to a pre-natal diagnosis of a malformation, the actual risk of malformations would only be revealed if spontaneous and induced abortions were included. No such association was observed with organic solvent exposure in our previous study of spontaneous abortion among nickel-exposed workers in Mončegorsk, but a weak risk could not be ruled out [41]. The incidence of induced abortions in Mončegorsk due to a pre-natal diagnosis of a severe malformation (including chromosomal) was 17/1000 births in the period 2000-2004, while the incidence of late spontaneous abortions (12-27 weeks of gestation) was 5/1000 births (based on clinical figures obtained from the hospital in Mončegorsk).

Due to the rareness of the specific malformations, studies tend to collapse them into one outcome to gain numbers [6,8,12]. This weakens the specificity of the statistical analysis because of the heterogeneity of the malformations. Mixed exposure (to more than one organic solvent) constitutes a similar limitation. Studies tend to treat them as a single group since they all cross the placenta easily and tend to be used as mixtures, or interchangeably in occupational settings. Since different malformations have different aetiologies, and perhaps solvent specificities, an overall increase in risk due to exposure to one specific organic solvent would be low. Thus, the likelihood of detecting a change in overall risk can be expected to be higher when exposure is to several solvents, and this pertains to our study.

Another possible limitation relates to confounding causative factors not adjusted for. However, diabetes mellitus and relevant infectious diseases were rather rare in the study population [25]. The same was observed for alcohol abuse, which was highly correlated with observed smoking habits. *Post hoc *inclusions of diabetes and alcohol abuse in the regression models did not change the parameter estimates for the exposure variable. The peri-conceptional folate status of the women in Mončegorsk, as in the RF, was not known, and prophylactic measures involving folate did not exist during most of the study period. However, there were no neural tube defects in the exposed group, which suggests that such participants did not have a poorer folate status than the non-exposed.

All confounders included were positively associated with the outcome. The year of birth (OR = 1.08; 95% C.L.: 1.05, 1.12), which was included in the model to adjust for structural and systematic changes over time, indicates that the prevalence of malformations increased slightly over time. Concominantly, the minimum duration of clinical observation before discharge was gradually shortened (from 7 to 3-4 days), and the number of women working as painters diminished from the late 1980s on. The regulations concerning work that may be hazardous during pregnancy remained the same throughout the study period. It is to be noted that the occupational regulations pertaining to hazardous substances during pregnancy remained more or less the same throughout the study period. On the other hand, the relative use of the different solvents used has changed over the years. For example, it is known to the authors that the use of benzene by the painters ceased after the dissolution of the Soviet Union, and that of glycol ethers appears to have increased. There have also been changes in the clinical practises over the years, with some improvements in detection and diagnosis of malformations during the perinatal period. These developments might have contributed to the increased prevalence over time.

The prospective nature of the exposure and outcome data was a strength of our study. The results were therefore not affected by selection bias based on exposure, nor by recall bias as in case-control studies. In addition, the size of the study cohort facilitated the inclusion of a homogenous group of workers from a well-defined population, permitting the univariate analyses of subgroups of congenital anomalies. An association between paternal exposure to organic solvents and an increased risk of malformations has only been been reported for neural tube defects [42]. Such an association was therefore unlikely at play in the present study.

In the exposed group, we included only women in jobs known to entail high daily exposure to a mixture of organic solvents. However, exposure misclassification remained a possibility. It constitutes a weakness in all studies of maternal occupational exposures that deduce exposure from maternal job title [43]. A mandatory job transfer for women in potentially hazardous jobs usually took place after the first antenatal visit, and the mean gestational age for this was 14 weeks (59% before 13 completed weeks). The critical window of suceptibility for impaired organ development varies between organs, but tends to be periconceptional and within a short period of organogenesis. Thus, some of the women in exposed jobs may have been absent from work during the criticial period, or were transferred to another job prior to the critical time for some types of malformations. Such a misclassification of exposure would have biased our findings towards the null, and impacted the diffent categories of malformations differently.

## Conclusion

There appeared to be a higher risk of malformations of the circulatory system and genital organs at birth among newborns to women in occupations with solvent exposure during early pregnancy (predominantly employed as painters). However, the findings were not statistically conclusive. Considering that these two categories of malformations are not readily diagnosed perinatally with late manifestations a possibility, the difference in prevalence between the exposed and unexposed may have been underestimated.

## Competing interests

The authors declare that they have no competing interests.

## Authors' contributions

All three authors participated in defining the exposed and non-exposed groups, and in revising the manuscript. AV conducted the statistical analyses, and drafted the original manuscript. LT set up the database, and EN critically edited the final draft. Each author approved the final version.

## Endnotes

^a ^Plasterers, hairdressers, manicurists, pedicurists, decorators, electricians, dry cleaners and cleaners; and petroleum, agriculture, shoe factory and laboratory workers.

^b ^Welders, nurses and industrial machine operators; gas, electronic, building, construction, textile, chemical and military workers.

## Pre-publication history

The pre-publication history for this paper can be accessed here:

http://www.biomedcentral.com/1471-2393/11/83/prepub
